# Neuronal loss drives differentially expressed protein‐pathways in the PSP globus pallidus

**DOI:** 10.1002/ctm2.1280

**Published:** 2023-07-10

**Authors:** Fiona Dick, Gard S. Johanson, Charalampos Tzoulis

**Affiliations:** ^1^ Neuro‐SysMed Center Department of Neurology Haukeland University Hospital Bergen Norway; ^2^ K.G Jebsen Center for Translational Research in Parkinson's Disease University of Bergen Bergen Norway; ^3^ Department of Clinical Medicine University of Bergen Bergen Norway

**Keywords:** mitochondria, neurodegeneration, progressive supranuclear palsy, proteomics, ribosomes

## 1 COMMENTARY

Jang et al. recently reported differentially expressed protein profiles in bulk‐tissue samples from the globus pallidus (GP) of individuals with progressive supranuclear palsy (PSP). Employing mass spectrometry‐based proteomics, the authors showed that PSP is characterized by downregulation of mainly mitochondrial proteins and concluded that this is potentially linked to the pathogenesis of the disease.^1^

Since the GP is characterized by severe neurodegeneration in PSP,^2^ we wondered whether the reported findings may reflect altered cell composition in the samples, rather than regulatory disease‐processes. We have previously shown that a similar reduction in mitochondrial transcripts is secondary to neurodegeneration in Parkinson's disease (PD).^3^

To address this question, we estimated the cell‐type composition of the samples using marker gene profiles (MGPs) for astrocytes, microglia, neurons and oligodendrocytes and repeated the analyses adjusting for cell‐type composition^4^ (see “Supplementary Material” for description of methods).

**FIGURE 1:**
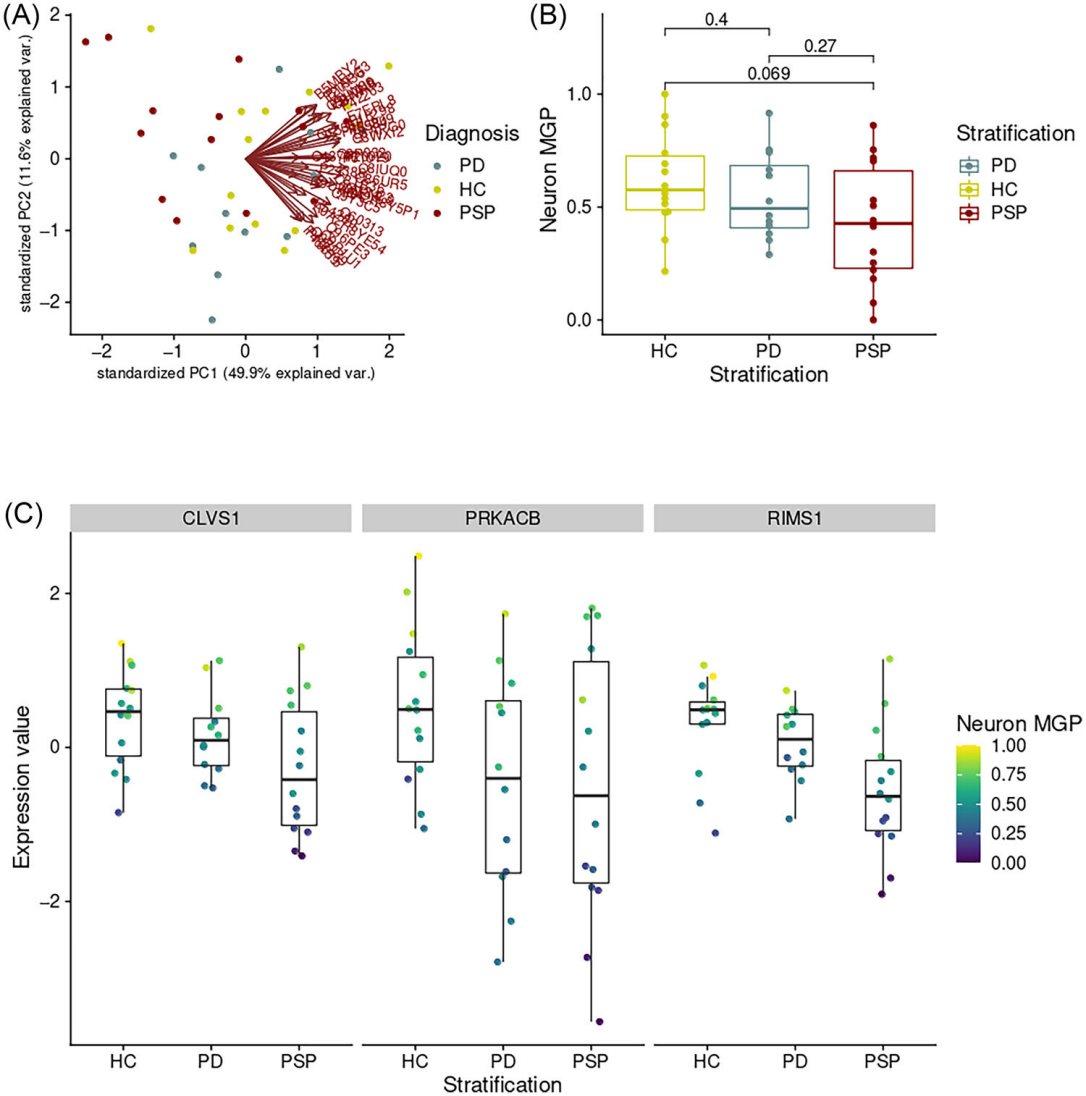
Decreased neuronal estimates in progressive supranuclear palsy (PSP). (A) Principal component analysis (PCA) plot based on neuronal marker gene expression. Data points represent individuals in the first (x‐axis) and second (y‐axis) principal component (PC) space. Disease state is indicated by color. Brown arrows indicate loading vectors, where small angles between them indicate correlation (coexpressed proteins). (B) Neuronal marker gene profile (MGP) (y‐axis) shown per group (x‐axis). Color highlights disease state. Differences between groups were tested using Wilcoxon test (*p*‐value is displayed). (C) Protein expression values (y‐axis) for three selected proteins (facets) plotted separately for each group (x‐axis). Color indicates neuronal MGP, where higher values indicate higher cell estimates.

The neuronal marker protein expression visually indicated a separation of PSP samples from the remaining individuals (Figure 1A). Cell estimates were decreased in PSP compared to controls (Figure 1B). Further supporting this, the top three neuronal marker proteins exhibited decreased expression in PSP compared to Healthy controls (HC) (Figure 1C).

We investigated the influence of cell composition on the entire protein dataset using principal component (PC) analysis and linear models to test for significant association between the first PC, representing the general expression pattern, and cell‐type estimates, while accounting for age, sex and batch. In the first linear model, we found a significant (*p* = 0.047) association between PC1 and the disease state. However, the association became non‐significant (*p* = 0.45) when cell‐type estimates were included in the model. Conversely, we observed a highly significant association (*p* < .01) of PC1 with the neuronal MGP, indicating that the main axis of variation can be explained by cell composition.

**FIGURE 2:**
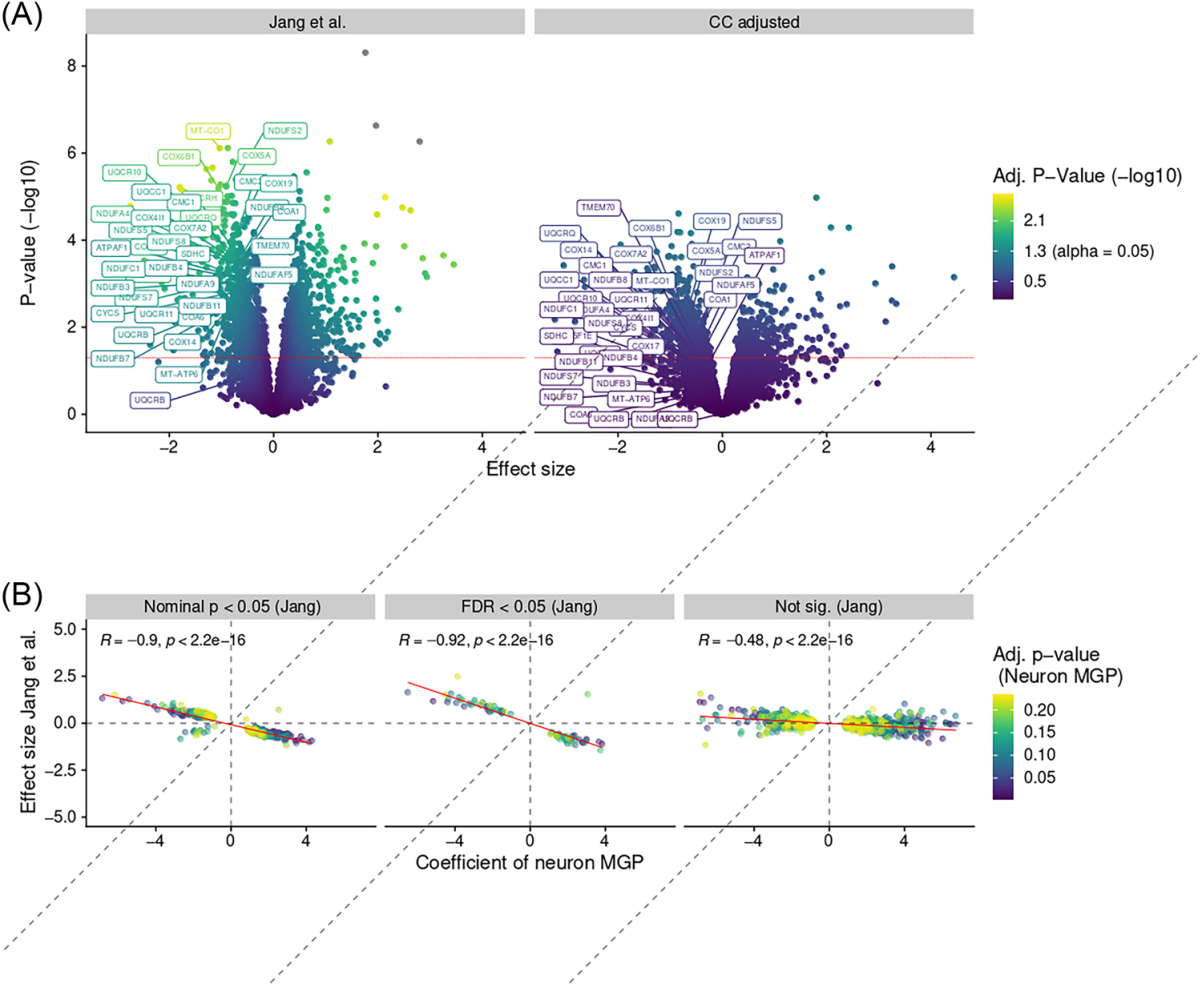
Effect size and significance comparison. (A) Volcano plot showing the results of the original work (left) and the cell‐adjusted model (right). Proteins (datapoints) are placed according to their effect size (x‐axis) and nominal significance (y‐axis). Nominal significance threshold at *α* = 0.05 is indicated as red dashed line. Color indicates FDR adjusted *p*‐value. Gene symbol labels are displayed for all proteins part of the MRC. (B) Correlation of effect size for neuronal marker gene profile (MGP) coefficient extracted from the cell adjusted model (x‐axis) and the effect size (progressive supranuclear palsy [PSP] vs. HC) reported in the original work (y‐axis) for proteins nominally significant for the neuronal MGP coefficient (i.e., where protein expression is significantly associated with the neuronal MGP). A positive MGP coefficient indicates that protein expression increases (or decreases) as neuronal estimates increase (or decrease). Negative MGP coefficients indicate increased (or decreased) protein expression with decreased (or increased) neuronal estimates. Proteins are grouped by significance as reported in the original work and colored by FDR adjusted significance of the neuronal MGP. Correlation indicated as Pearson correlation. Red line indicates linear fit y–x.

Differential protein expression (DPE) without cell composition adjustment (model I) identified *N* = 54 significant (False discovery rate (FDR) < 0.05) proteins, which were enriched for mitochondrial‐related pathways, as reported.^1^ DPE analysis with cell adjustment (model II) revealed no significant proteins after multiple testing correction. To specifically explore the effect of cell composition on Mitochondrial respiratory chain (MRC) proteins, which constituted the strongest signal reported by Jang et al., we selected MRC proteins which were reported significant (*q*‐value < 0.05) in the original work and assessed the effects of adjusting for cell‐type composition on significance and effect size (Figure 2A). We observed a clear loss of significance with many falling under the nominal significance threshold. Thus, the mitochondrial signal observed in model I is attenuated when accounting for cell‐type composition bias.

Finally, we compared the effect size (PSP vs. HC) reported in the original paper, with the effect size of the neuronal MGP, for all proteins that showed a significant association between expression level and neuronal MGP in model II. We found that proteins reported as differentially expressed in the original work showed a strong correlation (Pearson |r| > 0.9) between their effect size and the coefficient of the neuronal MGP (Figure 2B). For example, downregulated proteins in PSP showed a positive MGP coefficient, indicating that protein expression correlates positively with cell estimates. This is consistent with neuronal loss driving the observed results.

Our results show that the pathways identified to be associated with PSP^1^ are most likely driven by neuronal loss, rather than by disease‐specific regulatory changes. This is expected as neurons are enriched in mitochondria compared to other cell types in the brain. Consequently, neuronal loss presents as an apparent downregulation of mitochondrial proteins in bulk‐tissue analyses.^2^, ^3^, ^5^

Circumventing this confounder poses a major challenge to neurodegeneration omics. One approach, which we attempted here, is to adjust for estimated cell‐type composition. Our analyses confirm the expected neuronal loss in the GP of the PSP patients. Moreover, following adjustment for cell‐type composition, we find no significantly differentially expressed proteins between PSP and HC.

It should be stressed that our results do not necessarily refute that the GP may exhibit altered protein expression, including the downregulation of mitochondrial pathways, in PSP. However, due to the profound confounding effect of neuronal loss on protein expression in this region, it is not possible to confidently disentangle in‐cell changes from changes driven by cell‐type composition in the bulk GP samples studied by Jang et.al. A decrease in the abundance of mitochondrial respiratory chain complexes of the magnitude suggested by the original findings should be readily detectable by cell‐specific methods, such as immunohistochemistry, as previously described.^6^ We would therefore recommend that this finding is validated by this approach.

In conclusion, while we consider the study by Jang et al. to be an important contribution of novel data to the field, we advocate caution when interpreting the results. Based on the reported data, it is not possible to nominate novel disease pathways in PSP. The observed differences in protein expression profile are most likely attributed to changes in the cell composition of the GP in PSP.

## 2 METHODS

DPE was performed using functions lmFit and eBayes from the limma R‐package.^7^ To test for differences between PSP and controls, we designed the following models without and with cell estimates: I) “∼ Age + Batch + Sex + Stratification” and II) “∼ Age + Batch + Sex + Neurons + Oligodendrocytes + Stratification.” Before DPE, we performed surrogate variable (SV) analysis (R‐package sva^8^) with I) as the base‐model to investigate the correlation between SV (unexplained bias) and cell‐type estimates. In a second iteration, we included the most correlating cell estimates with SV, neurons, and oligodendrocytes, into the base‐model (II) to ensure that remaining bias is not explained by cell composition. Results of the SV analysis indicated neurons and oligodendrocytes as the main contributors to the observed bias. We decided to include the MGPs for these in our cell composition‐adjusted model (model II).

On significant (FDR < 0.05) proteins, we performed overrepresentation analysis (R‐package WebGestaltR^9^) using a nonredundant subset of GO pathways. Estimation of MGPs was performed as described,^3^, ^4^ using cell‐type markers from Kelley, et al.^10^ and Velmeshev, et al.^11^

Performance of marker genes was warranted by sufficiently high explained variance (%) and good agreement among markers (Figure S1).

## Supporting information

Supporting Information
