# Supplementary material for: Neuronal loss drives differentially expressed protein‐pathways in the PSP globus pallidus
Source: Clin Transl Med. 2023 Jul 10;13(7):e1280. doi: 10.1002/ctm2.1280 (PMC10331573; doi:10.1002/ctm2.1280)
Supplement: Supplementary file 1 — Supporting Information [file CTM2-13-e1280-s001.docx]

**Supplementary material**

**Methods**

Differential protein expression (DPE) was performed using functions *lmFit* and *eBayes from* the *limma* R-package^7^. To test for differences between PSP and controls we designed the following models without and with cell estimates: I) “~ Age + Batch + Sex + Stratification” and II) “~ Age + Batch + Sex + Neurons + Oligodendrocytes + Stratification”. Before DPE we performed surrogate variable (SV) analysis (R-package *sva*^8^) with I) as the base-model to investigate the correlation between SV (unexplained bias) and cell-type estimates. In a second iteration, we included the most correlating cell estimates with SV, neurons and oligodendrocytes, into the base-model (II) to ensure that remaining bias is not explained by cell composition. Results of the SV analysis indicated neurons and oligodendrocytes as the main contributors to the observed bias. We decided to include the MGPs for these in our cell composition-adjusted model (model II)

On significant (FDR<0.05) proteins we performed overrepresentation analysis (R-package *WebGestaltR*^9^ ) using a non-redundant subset of GO pathways. Estimation of MGPs was performed as described^3,4,^ using cell-type markers from Kelley K, et al.^10^ and Velmeshev D, et al.^11^

Performance of marker genes was warranted by sufficiently high explained variance (%) and good agreement among markers (Figure S1).


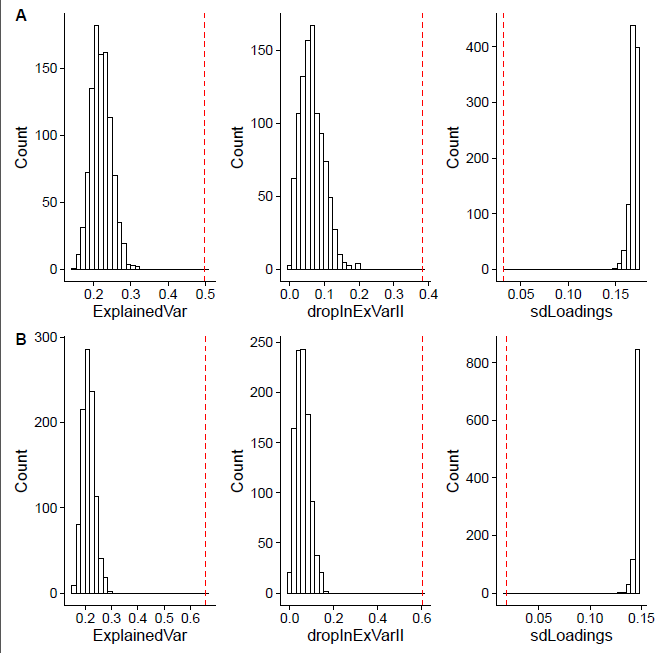


**Figure S1**

**Performance of MGP**

Results of principal component (PC) analysis on a randomly chosen subset of proteins (N = 1000 permutations). Displayed from left to right are: the distribution of explained variance of PC1, the drop of explained variance between PC1 and PC2, and the standard deviation of the loadings of PC1. Respective statistics for the principal component analysis on the selected marker genes are indicated in red for **A:** Neurons and **B:** Oligodendrocytes.
